# The genome sequence of a drosophilid fruit fly, *Chymomyza fuscimana *(Drosophilidae) (Zetterstedt, 1838)

**DOI:** 10.12688/wellcomeopenres.20122.1

**Published:** 2023-10-18

**Authors:** Darren J. Obbard, Wellcome Sanger Institute Tree of Life programme, Wellcome Sanger Institute Scientific Operations: DNA Pipelines collective, Tree of Life Core Informatics collective, Darwin Tree of Life Consortium

**Affiliations:** 1 Institute of Ecology and Evolution, The University of Edinburgh, Edinburgh, Scotland, UK

**Keywords:** Chymomyza fuscimana, drosophilid fruit fly, genome sequence, chromosomal, Diptera

## Abstract

We present a genome assembly from an individual male *Chymomyza fuscimana* (drosophilid fruit fly; Arthropoda; Insecta; Diptera; Drosophilidae). The genome sequence is 338.0 megabases in span. Most of the assembly is scaffolded into 5 chromosomal pseudomolecules, including the X and Y sex chromosomes. The mitochondrial genome has also been assembled and is 16.47 kilobases in length.

## Species taxonomy

Eukaryota; Metazoa; Eumetazoa; Bilateria; Protostomia; Ecdysozoa; Panarthropoda; Arthropoda; Mandibulata; Pancrustacea; Hexapoda; Insecta; Dicondylia; Pterygota; Neoptera; Endopterygota; Diptera; Brachycera; Muscomorpha; Eremoneura; Cyclorrhapha; Schizophora; Acalyptratae; Ephydroidea; Drosophilidae; Drosophilinae; Colocasiomyini; *Chymomyza*; *Chymomyza fuscimana* (Zetterstedt, 1838) (NCBI:txid1692350).

## Background

**Figure 1.**
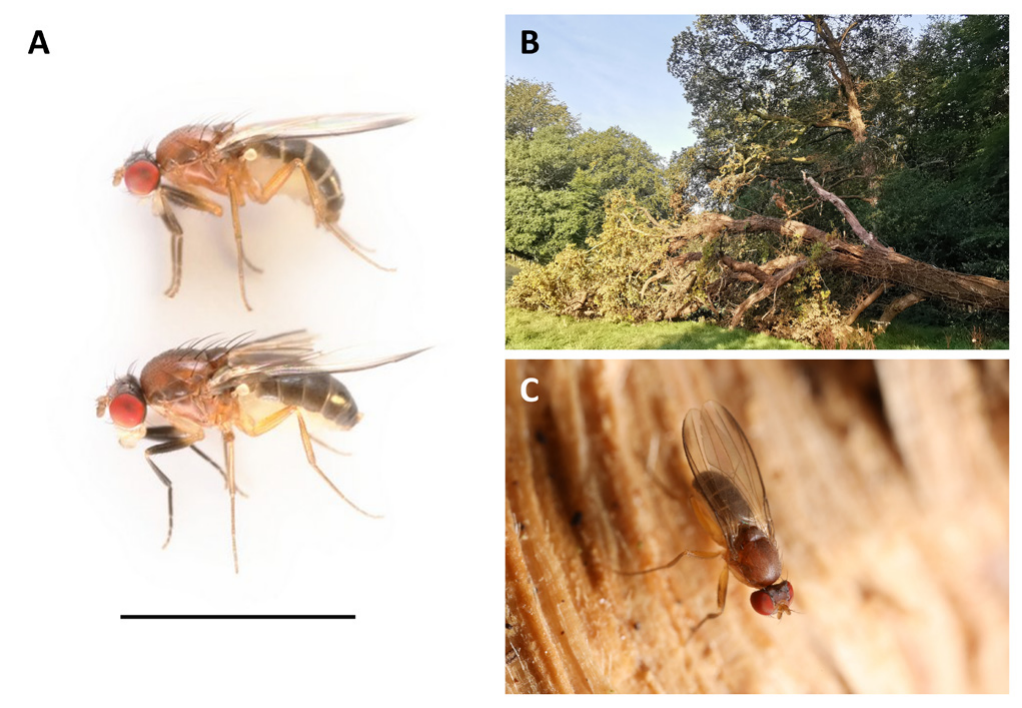
**A:** Male (above) and female (below) *Chymomyza fuscimana* presented with a 3 mm scale bar. **B:** The fallen oak from which the sequenced individuals were collected (Penns in the Rocks estate, East Sussex, England; 51.093N,0.1698E). **C:** Male fly on a freshly broken surface of the oak.

*Chymomyza fuscimana* (Zetterstedt, 1838) is a medium sized ( *ca.* 3.0–3.5 mm) drosophilid ‘fruit fly’ with striking red eyes and contrasting yellowish-brown and black body colouration ( Figure 1A). It is very distantly related to the laboratory model *Drosophila melanogaster* within the subfamily Drosophilinae ( Finet *et al.*, 2021), and is one of six species of *Chymomyza* recorded from Britain and Ireland ( Chandler, 2023). It is morphologically close to *Chymomyza distincta*, but without dissection can still be distinguished by the absence (in male *fuscimana*) of long white setulae on the inner side of the procoxa ( Bächli *et al.*, 2004).

As in many other species of *Chymomyza*, the larvae of *C. fuscimana* feed in fermenting sap under the bark of fallen and damaged trees, including both broad-leaved and coniferous species ( Burla, 1995; Burla, 1997; Rotheray & Robertson, 1998). The adults can often be seen on freshly exposed timber surfaces, including newly fallen trees, broken branches, and sawn logs and stumps ( Figure 1B and C) – where males display and compete by wing-waving and raising their front legs ( Burla, 1995; Burla, 1997; Chandler, 1978; Perry, 2019).

*Chymomyza fuscimana* is broadly distributed across the northern Palearctic, from Ireland in the west to the far east of Russia, and from Greece in the south to the north of Finland ( Bächli, 2023). Although relatively few records are available in the UK, adults appear to be most active between May and October ( GBIF Secretariat, 2023). The species is not reported to be threatened, and it seems likely that the scarcity of UK records reflects the challenge of identification (e.g. Basden, 1961), the failure of these flies to come to baits, and/or the transience and accessibility of freshly exposed timber ( Burla, 1995; Chandler, 1978).

Here we present a chromosomally complete genome sequence for *Chymomyza fuscimana*, derived from the DNA and RNA of three adult male flies collected from a fallen oak tree on the Penns in the Rocks estate, East Sussex, as part of the Darwin Tree of Life Project. This genome sequence will help to resolve relationships among the Drosophilidae and will further build on the value of this family as a model clade for comparative genomics and molecular evolution. This project is a collaborative effort to sequence all named eukaryotic species in the Atlantic Archipelago of Britain and Ireland.

## Genome sequence report

The genome was sequenced from one male *Chymomyza fuscimana* ( Figure 1) collected from Penns in the Rocks Estate, East Sussex, UK (51.09, 0.17). A total of 65-fold coverage in Pacific Biosciences single-molecule HiFi long reads was generated. Primary assembly contigs were scaffolded with chromosome conformation Hi-C data. Manual assembly curation corrected 69 missing joins or mis-joins and removed 5 haplotypic duplications, reducing the assembly length by 0.55 % and the scaffold number by 76.81%, and increasing the scaffold N50 by 60.81%.

**Figure 2.**
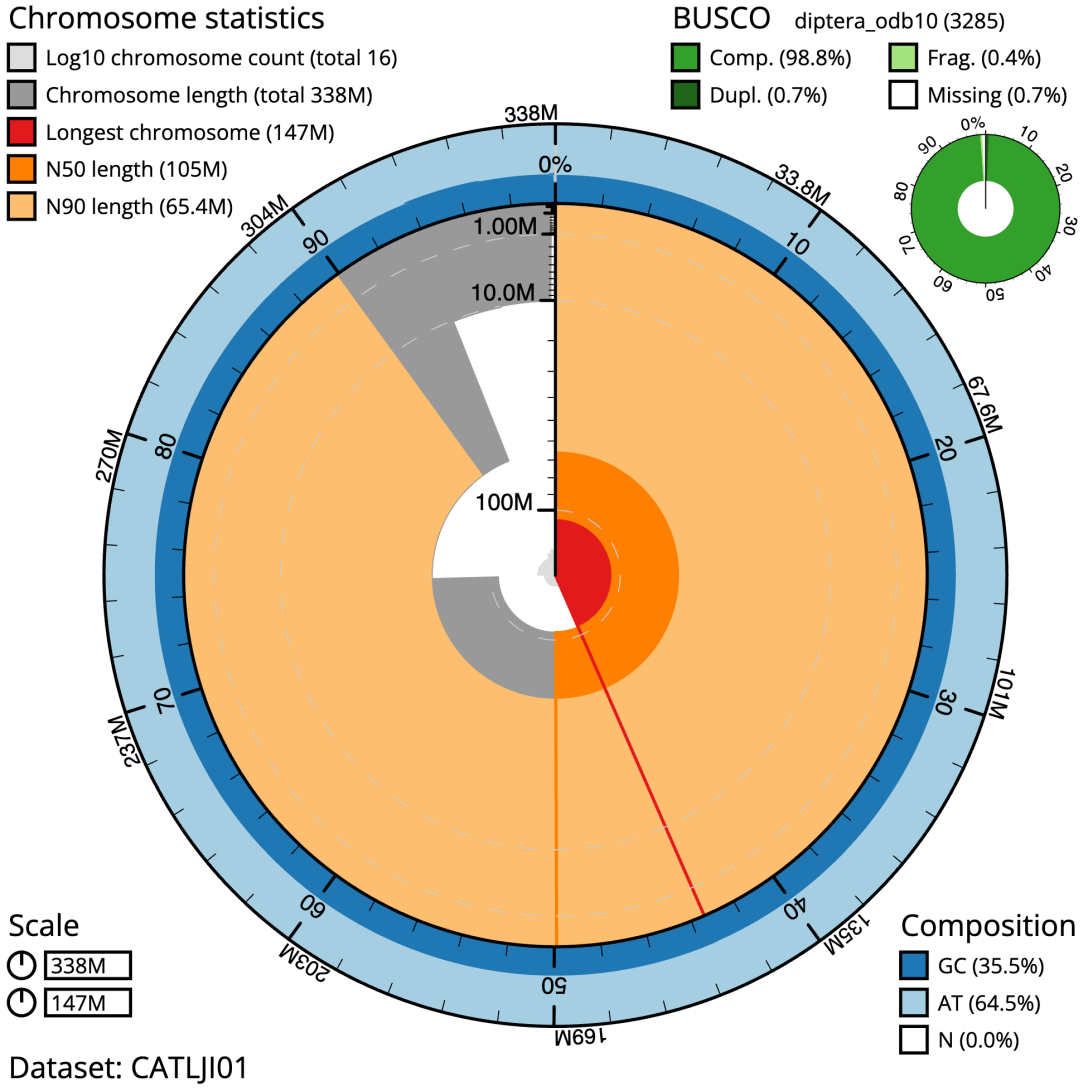
Genome assembly of *Chymomyza fuscimana*, idChyFusc2.1: metrics. The BlobToolKit Snailplot shows N50 metrics and BUSCO gene completeness. The main plot is divided into 1,000 size-ordered bins around the circumference with each bin representing 0.1% of the 337,977,858 bp assembly. The distribution of scaffold lengths is shown in dark grey with the plot radius scaled to the longest scaffold present in the assembly (146,831,255 bp, shown in red). Orange and pale-orange arcs show the N50 and N90 scaffold lengths (104,850,594 and 65,373,579 bp), respectively. The pale grey spiral shows the cumulative scaffold count on a log scale with white scale lines showing successive orders of magnitude. The blue and pale-blue area around the outside of the plot shows the distribution of GC, AT and N percentages in the same bins as the inner plot. A summary of complete, fragmented, duplicated and missing BUSCO genes in the diptera_odb10 set is shown in the top right. An interactive version of this figure is available at https://blobtoolkit.genomehubs.org/view/Chymomyza%20fuscimana/dataset/CATLJI01/snail.

**Figure 3.**
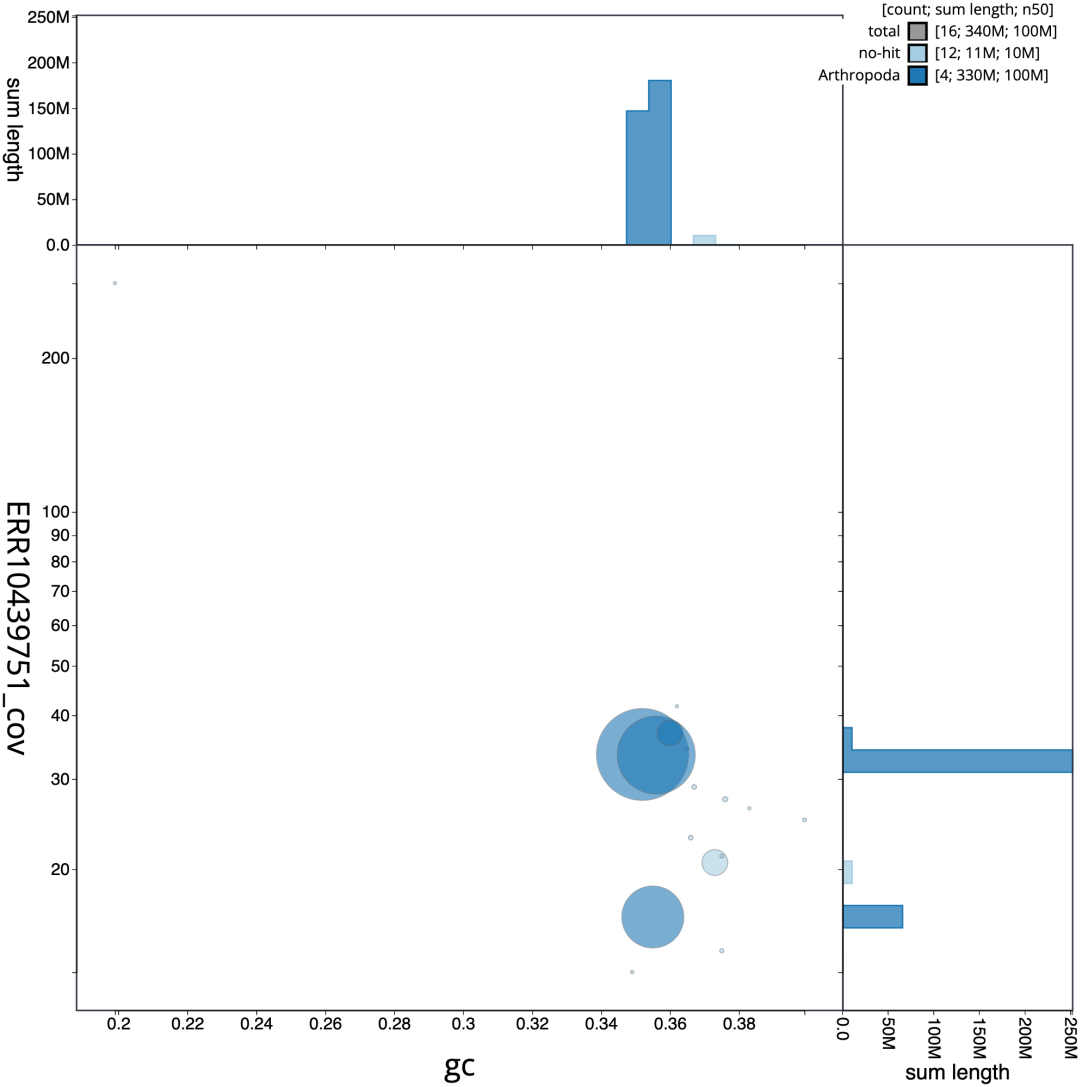
Genome assembly of *Chymomyza fuscimana*, idChyFusc2.1: BlobToolKit GC-coverage plot. Scaffolds are coloured by phylum. Circles are sized in proportion to scaffold length. Histograms show the distribution of scaffold length sum along each axis. An interactive version of this figure is available at https://blobtoolkit.genomehubs.org/view/Chymomyza%20fuscimana/dataset/CATLJI01/blob.

**Figure 4.**
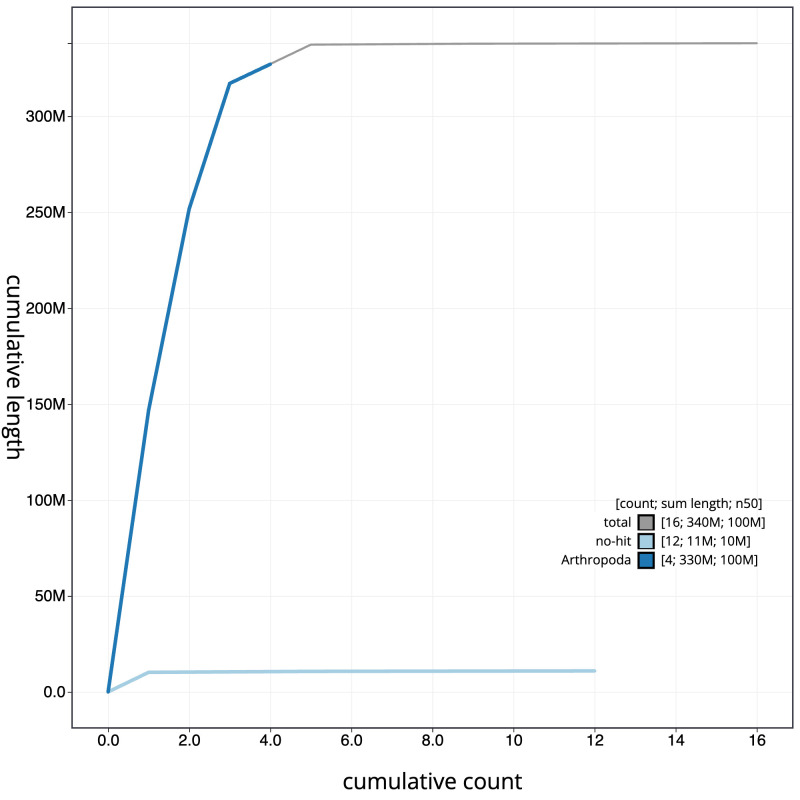
Genome assembly of *Chymomyza fuscimana*, idChyFusc2.1: BlobToolKit cumulative sequence plot. The grey line shows cumulative length for all scaffolds. Coloured lines show cumulative lengths of scaffolds assigned to each phylum using the buscogenes taxrule. An interactive version of this figure is available at https://blobtoolkit.genomehubs.org/view/Chymomyza%20fuscimana/dataset/CATLJI01/cumulative.

**Figure 5.**
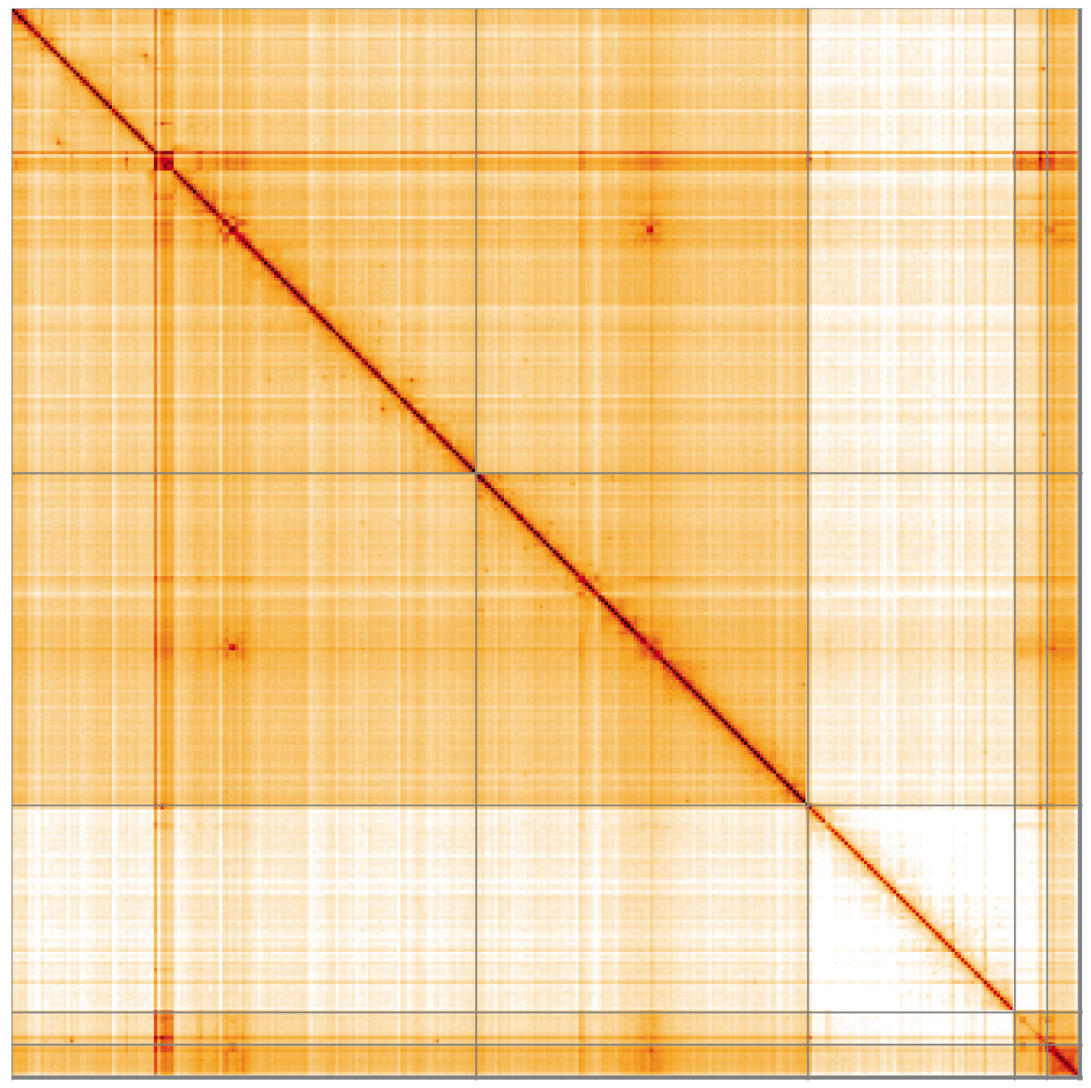
Genome assembly of *Chymomyza fuscimana*, idChyFusc2.1: Hi-C contact map of the idChyFusc2.1 assembly, visualised using HiGlass. Chromosomes are shown in order of size from left to right and top to bottom. An interactive version of this figure may be viewed at https://genome-note-higlass.tol.sanger.ac.uk/l/?d=cxKt_xm7TFqYAi236-3nRg.

The final assembly has a total length of 338.0 Mb in 15 sequence scaffolds with a scaffold N50 of 104.9 Mb ( Table 1). A summary of the assembly statistics is shown in Figure 2, while the distribution of assembly scaffolds on GC proportion and coverage is shown in Figure 3. The cumulative assembly plot in Figure 4 shows curves for subsets of scaffolds assigned to different phyla. Most (99.78%) of the assembly sequence was assigned to 5 chromosomal-level scaffolds, representing 3 autosomes and the X and Y sex chromosomes. Chromosome-scale scaffolds confirmed by the Hi-C data are named in order of size ( Figure 5; Table 2). The X and Y chromosome were identified by half read coverage, and chromosome 3 might be the equivalent of a *Drosophila* dot chromosome. While not fully phased, the assembly deposited is of one haplotype. Contigs corresponding to the second haplotype have also been deposited. The mitochondrial genome was also assembled and can be found as a contig within the multifasta file of the genome submission.

**Table 1.** Genome data for *Chymomyza fuscimana*, idChyFusc2.1.

Project accession data		
Assembly identifier	idChyFusc2.1	
Assembly release date	2023-05-03	
Species	*Chymomyza fuscimana*	
Specimen	idChyFusc2	
NCBI taxonomy ID	1692350	
BioProject	PRJEB57111	
BioSample ID	SAMEA12110601	
Isolate information	idChyFusc2, male: whole organism (DNA sequencing) idChyFusc1, male: whole organism (Hi-C scaffolding)	
Assembly metrics *		*Benchmark*
Consensus quality (QV)	62	*≥ 50*
*k*-mer completeness	100%	*≥ 95%*
BUSCO **	C:98.8%[S:98.1%,D:0.7%], F:0.4%,M:0.7%,n:3,285	*C ≥ 95%*
Percentage of assembly mapped to chromosomes	99.78%	*≥ 95%*
Sex chromosomes	X and Y	*localised homologous pairs*
Organelles	Mitochondrial genome assembled	*complete single alleles*
Raw data accessions		
PacificBiosciences SEQUEL II	ERR10439751	
HiC Illumina	ERR10446385	
Genome assembly		
Assembly accession	GCA_949987675.1	
*Accession of alternate haplotype*	GCA_949987665.1	
Span (Mb)	338.0	
Number of contigs	265	
Contig N50 length (Mb)	2.7	
Number of scaffolds	15	
Scaffold N50 length (Mb)	104.9	
Longest scaffold (Mb)	146.8	

* Assembly metric benchmarks are adapted from column VGP-2020 of “Table 1: Proposed standards and metrics for defining genome assembly quality” from ( Rhie *et al.*, 2021). ** BUSCO scores based on the diptera_odb10 BUSCO set using v5.3.2. C = complete [S = single copy, D = duplicated], F = fragmented, M = missing, n = number of orthologues in comparison. A full set of BUSCO scores is available at https://blobtoolkit.genomehubs.org/view/idChyFusc2.1/dataset/CATLJI01/busco.

**Table 2.** Chromosomal pseudomolecules in the genome assembly of *Chymomyza fuscimana*, idChyFusc2.

INSDC accession	Chromosome	Length (Mb)	GC%
OX465088.1	1	146.83	35.0
OX465089.1	2	104.85	35.5
OX465092.1	3	9.99	36.0
OX465090.1	X	65.37	35.5
OX465091.1	Y	10.2	37.5
OX465093.1	MT	0.02	20.0

The estimated Quality Value (QV) of the final assembly is 62 with *k*-mer completeness of 100%, and the assembly has a BUSCO v5.3.2 completeness of 98.8% (single = 98.1%, duplicated = 0.7%), using the diptera_odb10 reference set ( *n* = 3,285).

Metadata for specimens, spectral estimates, sequencing runs, contaminants and pre-curation assembly statistics can be found at https://links.tol.sanger.ac.uk/species/1692350.

## Methods

### Sample acquisition and nucleic acid extraction

*Chymomyza fuscimana* specimens were collected from the broken surface of a fallen oak in Penns in the Rocks Estate, East Sussex, UK (latitude 51.09, longitude 0.17) on 2021-09-07. The specimens were collected and identified by Darren Obbard (University of Edinburgh) and frozen from live (– 80°C). The specimen used for genome sequencing was specimen ID SAN00002034, ToLID idChyFusc2), while the specimen used for Hi-C scaffolding was specimen ID SAN00002033, ToLID idChyFusc1. A third specimen ID SAN00002035, ToLID idChyFusc3, was used to generate poly-A selected RNAseq data.

DNA was extracted at the Tree of Life laboratory, Wellcome Sanger Institute (WSI). The idChyFusc2 sample was weighed and dissected on dry ice. Tissue from the whole organism was disrupted using a Nippi Powermasher fitted with a BioMasher pestle. High molecular weight (HMW) DNA was extracted using the Qiagen MagAttract HMW DNA extraction kit. HMW DNA was sheared into an average fragment size of 12–20 kb in a Megaruptor 3 system with speed setting 30. Sheared DNA was purified by solid-phase reversible immobilisation using AMPure PB beads with a 1.8X ratio of beads to sample to remove the shorter fragments and concentrate the DNA sample. The concentration of the sheared and purified DNA was assessed using a Nanodrop spectrophotometer and Qubit Fluorometer and Qubit dsDNA High Sensitivity Assay kit. Fragment size distribution was evaluated by running the sample on the FemtoPulse system.

### Sequencing

Pacific Biosciences HiFi circular consensus DNA sequencing libraries were constructed according to the manufacturers’ instructions. DNA sequencing was performed by the Scientific Operations core at the WSI on a Pacific Biosciences SEQUEL II (HiFi) instrument. Hi-C data were also generated from the whole organism tissue of idChyFusc1 using the Arima2 kit and sequenced on the Illumina NovaSeq 6000 instrument.

### Genome assembly, curation and evaluation

Assembly was carried out with Hifiasm ( Cheng *et al.*, 2021) and haplotypic duplication was identified and removed with purge_dups ( Guan *et al.*, 2020). The assembly was then scaffolded with Hi-C data ( Rao *et al.*, 2014) using YaHS ( Zhou *et al.*, 2023). The assembly was checked for contamination and corrected as described previously ( Howe *et al.*, 2021). Manual curation was performed using HiGlass ( Kerpedjiev *et al.*, 2018) and Pretext ( Harry, 2022). The mitochondrial genome was assembled using MitoHiFi ( Uliano-Silva *et al.*, 2023), which runs MitoFinder ( Allio *et al.*, 2020) or MITOS ( Bernt *et al.*, 2013) and uses these annotations to select the final mitochondrial contig and to ensure the general quality of the sequence.

A Hi-C map for the final assembly was produced using bwa-mem2 ( Vasimuddin *et al.*, 2019) in the Cooler file format ( Abdennur & Mirny, 2020). To assess the assembly metrics, the *k*-mer completeness and QV consensus quality values were calculated in Merqury ( Rhie *et al.*, 2020). This work was done using Nextflow ( Di Tommaso *et al.*, 2017) DSL2 pipelines “sanger-tol/readmapping” ( Surana *et al.*, 2023a) and “sanger-tol/genomenote” ( Surana *et al.*, 2023b). The genome was analysed within the BlobToolKit environment ( Challis *et al.*, 2020) and BUSCO scores ( Manni *et al.*, 2021; Simão *et al.*, 2015) were calculated.

Table 3 contains a list of relevant software tool versions and sources.

**Table 3.** Software tools: versions and sources.

Software tool	Version	Source
BlobToolKit	4.1.7	https://github.com/blobtoolkit/blobtoolkit
BUSCO	5.3.2	https://gitlab.com/ezlab/busco
Hifiasm	0.16.1	https://github.com/chhylp123/hifiasm
HiGlass	1.11.6	https://github.com/higlass/higlass
Merqury	MerquryFK	https://github.com/thegenemyers/MERQURY.FK
MitoHiFi	2	https://github.com/marcelauliano/MitoHiFi
PretextView	0.2	https://github.com/wtsi-hpag/PretextView
purge_dups	1.2.3	https://github.com/dfguan/purge_dups
sanger-tol/genomenote	v1.0	https://github.com/sanger-tol/genomenote
sanger-tol/readmapping	1.1.0	https://github.com/sanger-tol/readmapping/tree/1.1.0
YaHS	yahs-1.2a.2	https://github.com/c-zhou/yahs

### Wellcome Sanger Institute – Legal and Governance

The materials that have contributed to this genome note have been supplied by a Darwin Tree of Life Partner. The submission of materials by a Darwin Tree of Life Partner is subject to the **‘Darwin Tree of Life Project Sampling Code of Practice’**, which can be found in full on the Darwin Tree of Life website here. By agreeing with and signing up to the Sampling Code of Practice, the Darwin Tree of Life Partner agrees they will meet the legal and ethical requirements and standards set out within this document in respect of all samples acquired for, and supplied to, the Darwin Tree of Life Project.

Further, the Wellcome Sanger Institute employs a process whereby due diligence is carried out proportionate to the nature of the materials themselves, and the circumstances under which they have been/are to be collected and provided for use. The purpose of this is to address and mitigate any potential legal and/or ethical implications of receipt and use of the materials as part of the research project, and to ensure that in doing so we align with best practice wherever possible. The overarching areas of consideration are:

•   Ethical review of provenance and sourcing of the material

•   Legality of collection, transfer and use (national and international)

Each transfer of samples is further undertaken according to a Research Collaboration Agreement or Material Transfer Agreement entered into by the Darwin Tree of Life Partner, Genome Research Limited (operating as the Wellcome Sanger Institute), and in some circumstances other Darwin Tree of Life collaborators.

## Data Availability

European Nucleotide Archive: *Chymomyza fuscimana*. Accession number PRJEB57111; https://identifiers.org/ena.embl/PRJEB57111. ( Wellcome Sanger Institute, 2023) The genome sequence is released openly for reuse. The *Chymomyza fuscimana* genome sequencing initiative is part of the Darwin Tree of Life (DToL) project. All raw sequence data and the assembly have been deposited in INSDC databases. The genome will be annotated using available RNA-Seq data and presented through the Ensembl pipeline at the European Bioinformatics Institute. Raw data and assembly accession identifiers are reported in Table 1.

## Author information

Members of the Wellcome Sanger Institute Tree of Life programme are listed here: https://doi.org/10.5281/zenodo.4783585.

Members of Wellcome Sanger Institute Scientific Operations: DNA Pipelines collective are listed here: https://doi.org/10.5281/zenodo.4790455.

Members of the Tree of Life Core Informatics collective are listed here: https://doi.org/10.5281/zenodo.5013541.

Members of the Darwin Tree of Life Consortium are listed here: https://doi.org/10.5281/zenodo.4783558.
