# Supplementary material for: The genome sequence of a drosophilid fruit fly, Chymomyza fuscimana (Drosophilidae) (Zetterstedt, 1838)
Source: Wellcome Open Res. 2023 Oct 18;8:477. [Version 1] doi: 10.12688/wellcomeopenres.20122.1 (PMC11294813; doi:10.12688/wellcomeopenres.20122.1)
Supplement: Peer review history

# Reviewer report: Reviewer response for version 1

**DOI:** 10.21956/wellcomeopenres.22284.r79982

**Published:** 2024-08-01

**Authors:** Tyler Scott Alioto

**Affiliations:** 1 Fundacion Centro Nacional de Analisi Genomico, Barcelona, Catalonia, Spain

The genome note submitted by Obbard *et al.* reports the genome sequence of the drosophilid fruit fly, *Chymomyza fuscimana*. The assembly is chromosome-scale and of high quality, meeting the minimum standards recommended by the Earth Biogenome Project. All protocols are appropriate and well-documented. All data conforms with FAIR principles, with read data and assemblies being available in the ENA. Blobtoolkit figures are interactive. Additional QC data are provided at https://tolqc.cog.sanger.ac.uk/, supplementing the figures published in the data note.

Are sufficient details of methods and materials provided to allow replication by others?

Yes

Is the rationale for creating the dataset(s) clearly described?

Yes

Are the datasets clearly presented in a useable and accessible format?

Yes

Are the protocols appropriate and is the work technically sound?

Yes

Reviewer Expertise:

genomics, genome assembly, gene prediction, annotation

I confirm that I have read this submission and believe that I have an appropriate level of expertise to confirm that it is of an acceptable scientific standard.

# Reviewer report: Reviewer response for version 1

**DOI:** 10.21956/wellcomeopenres.22284.r71587

**Published:** 2024-02-01

**Authors:** Bernard Moussian

**Affiliations:** 1 University of Tübingen, Tübingen, Germany

The genome of a male of the drosophila fly

*Chymomyza fuscimana *presented. The sequence is of high quality. For instance the Busco value is 98,8%. Only little unclear sequence information persists. For more clarity for the reader, I have two points:

 - in the abstract, the author writes "most of the assembly...". Information on the portion not being assembled should be provided.

 - in the genome sequence report, the author states that <"5 haplotypic duplications" were removed: was this a technical or a biological problem?

Are sufficient details of methods and materials provided to allow replication by others?

Yes

Is the rationale for creating the dataset(s) clearly described?

Yes

Are the datasets clearly presented in a useable and accessible format?

Yes

Are the protocols appropriate and is the work technically sound?

Yes

Reviewer Expertise:

Insect biology

I confirm that I have read this submission and believe that I have an appropriate level of expertise to confirm that it is of an acceptable scientific standard.
